# Comparative study of the three-dimensional genomes of granulosa cells in germinal vesicle and metaphase II follicles

**DOI:** 10.3389/fgene.2024.1480153

**Published:** 2024-11-20

**Authors:** Rurong Mao, Zhongkun Cai, Tao Wang, Yan Li, Shilin Tian, Diyan Li, Penghao Li

**Affiliations:** ^1^ Jinxin Research Institute for Reproductive Medicine and Genetics, Sichuan Jinxin Xi’nan Women’s and Children’s Hospital, Chengdu, China; ^2^ Antibiotics Research and Re-evaluation Key Laboratory of Sichuan Province, Sichuan Industrial Institute of Antibiotics, School of Pharmacy, Chengdu University, Chengdu, China; ^3^ Sichuan Key Laboratory of Conservation Biology on Endangered Wildlife, Chengdu Research Base of Giant Panda Breeding, Chengdu, Sichuan, China; ^4^ Global Product Center, Novogene Bioinformatics Institute, Beijing, China; ^5^ Assisted Reproductive Center, Yunnan Jinxin Jiuzhou Hospital, Kunming, Yunnan, China

**Keywords:** three-dimensional genome organization, granulosa cells, GV follicles, MII follicles, TAD, chromatin compartments

## Abstract

**Introduction:**

Follicle development is a critical process in the female reproductive system, with significant implications for fertility and reproductive health. Germinal vesicle (GV) oocytes are primary oocytes that are arrested in the dictyate stage, also known as the diplotene stage of meiotic prophase I. Metaphase II (MII) is the stage at which the oocyte is typically retrieved for assisted reproductive technologies such as *in vitro* fertilization (IVF). The granulosa cells play a pivotal role in follicle development processes. 3D chromatin organization is a fundamental aspect of cellular biology that has significant implications for gene regulation and cellular function.

**Methods:**

In this study, we investigated 3D chromatin organization in granulosacells from GV and MII follicles, which is essential for understanding the regulatory mechanisms governing oocyte development.

**Results:**

The results revealed distinct compartmentalization patterns,including stable genomic regions and transitions during oocyte maturation. Notably, there was a significant shift in functional gene activation, particularly in processes related to hormone metabolic pathways. Furthermore, alterations in topologically associating domains (TADs) were observed, with differential expression observed in genes that are involved in crucial biological processes. The analysis also identified a subset of genes with altered promoter-enhancer interactions (PEIs), reflecting a regulatory shift in gene expression related to reproductive processes.

**Discussion:**

These findings provide valuable insights into 3D genome organization in granulosa cells with implications for reproductive health and the development of assisted reproductive technologies. Understanding spatial genome organization at different stages of follicular development may help realize novel strategies for enhancing success rates in assisted reproductive technologies.

## 1 Introduction

Female infertility presents itself as a multifaceted health concern, resulting from single or multiple factors that hinder reproductive capabilities. Around 2%–10.5% women between the ages of 20 and 44 are affected (Wang et al., 2024b). The normal development of follicles determines the reproductive outcome. Folliculogenesis is a complex process that requires the integration of autocrine, paracrine and endocrine factors and tightly regulated interactions between granulosa cells and oocytes to enable the growth and survival of healthy follicles (Simon et al., 2020). Follicle development is a complex process that involves several distinct stages, such as primordial, primary, secondary, antral and post-ovulatory follicles (Zhang et al., 2018). The formation of the primordial follicle begins once the oocytes, derived from primordial germ cells (PGCs), detach from the germline cysts that divide synchronously. During fetal development, PGCs begin the process of meiosis. However, after the pairing and recombination of homologous chromosomes, they pause at the diplotene stage of prophase I of meiosis (MI). The resumption of meiosis from the prophase of MI is marked by the morphological change where the oocyte’s nuclear envelope dissolves, an event commonly referred to as “germinal vesicle breakdown” (GVBD). After GVBD and the completion of MI, the oocyte proceeds into meiosis II, bypassing a distinct S-phase, and halts at metaphase II (MII) until it is fertilized (Pan and Li, 2019). Within the intricate microenvironment of the ovarian follicle, granulosa cells play a fundamental role in supporting oocyte development and maturation (Li et al., 2022b). The granulosa cells surrounding the oocyte undergo profound morphological and functional changes during folliculogenesis (Eppig et al., 1997). Recent single-cell transcriptome studies have shown that granulosa cells can be divided into multiple cell groups during follicular development, with different functions (Leng et al., 2024; Wang et al., 2024a). These changes are associated with intricate molecular events, including alterations in gene expression patterns (Zhang et al., 2018). Oocyte–granulosa cell interactions exhibit stage- and species-specific patterns (Zhang et al., 2018).

In the eukaryotic cell nucleus, genomic DNA is highly folded and spatially organized into a hierarchy of 3D structures (Yu and Ren, 2017), which is crucial for the regulation of transcription (Hnisz et al., 2016). The exploration of 3D genome organization relies heavily on techniques such as Hi-C, which enables the mapping of chromatin interactions across the genomes of different species (Li et al., 2022a; Liu et al., 2023) or follicle developmental stages (Li et al., 2022b). Corresponding results include the identification of distinct chromatin compartments, topologically associated domains (TADs) (Lieberman-Aiden et al., 2009b), and long-range chromatin interactions (Paulsen et al., 2019) that are characteristic of each developmental stage. Changes in the three-dimensional architecture of the genome, such as the boundaries of topologically associated domains and the formation of chromatin loops, have been demonstrated to result in the activation of oncogenes and the advancement of cancer (Li et al., 2019; Animesh et al., 2021).

The transition from the germinal vesicle (GV) to the metaphase II (MII) stage involves dynamic changes in gene expression (Ntostis et al., 2021). They reported that during the GV to MII oocyte transition, 5,538 genes were differentially expressed in young maternal age (YMA) oocytes and 7,527 in advanced maternal age (AMA) oocytes, exhibiting a 36% increase in the number of differentially expressed genes in AMA oocytes (Ntostis et al., 2021). Considering the important role of granulosa cells in the development of oocytes, we believe that a dynamic reorganization of the chromatin landscape of granulosa cells during folliculogenesis would also occur. To elucidate the mechanisms driving the observed changes in 3D genome organization, we aimed to elucidate the dynamic changes in chromatin architecture in GV and MII granulosa cells.

## 2 Materials and methods

### 2.1 Isolation of human granulosa cells (GCs)

The GCs were collected from two patients (Supplementary Table S1). After oocyte retrieval under B-ultrasound monitoring, the obtained cumulus-oocyte complex (COC) was transferred to well 1 (300 μL, 80 U/mL hyaluronidase (ART-4007-A, SAGE, United States), 300 μL G-MOPS™ PLUS (10,130, Vitrolife, Sweden), and 500 μL mineral oil (ART-4008-5P, SAGE, United States) of the four-well plate using a pipette and repeatedly aspirated until the granulosa cells around the COC were separated from the oocyte and the aspiration time did not exceed 30 s. The oocytes were transferred to well 2 (500 μL of G-MOPS™ PLUS, 500 μL of mineral oil) of the four-well plate, rinsed 3–4 times, and then transferred to well 3 (500 μL of G-MOPS™ PLUS, 500 μL of mineral oil) of the four-well plate. After removing the granulosa cells around the oocyte, the oocyte was removed, and all granulosa cells were collected in a 200 μL tube.

### 2.2 Single-cell cDNA library construction and analysis from granulosa cells

Ten randomly selected granulosa cells from each stage were quickly transferred to lysis buffer using a mouth pipette. We next used Smart-Seq2 to perform single-cell transcriptome amplification with a few modifications. Briefly, single cells were sorted into cell lysis buffer containing 0.1 μL of RNase inhibitor (Clontech), 1.9 μL of Triton X-100 solution (1%), 1 μL of dNTP mix (10 mM), and 1 μL of oligo-dT primer (5 μM). Reverse transcription was performed with 0.5 μL of SuperScript II reverse transcriptase (200 U/μL, Invitrogen), 0.25 μL of RNase inhibitor (40 U/μL, Clontech), 2 μL of Superscript II First-Strand Buffer (5×, Invitrogen), 0.5 μL of DTT (0.1 M, Invitrogen), 2 μL of Betain (5 M, Sigma), 0.06 μL of MgCl2 (1 M, Sigma), and 0.1 μL of TSO (100 μM). Reverse transcription was carried out at 25 °C for 5 min, 42°C for 60 min, 50°C for 30 min and 72°C for 10 min. PCR preamplification was performed using KAPA HiFi HotStart Ready MIX (KAPA Biosystems) with 22 cycles of PCR, and the IS PCR primer was reduced to 50 nM (4 cycles at 98°C for 20 s, 65°C for 30 s, and 72°C for 5 min, followed by 18 cycles at 98°C for 20 s, 67 °C for 15 s, and 72°C for 5 min, with a final cycle at 72°C for 5 min). Subsequently, the amplified samples were purified twice with 0.8X AMPure XP beads (Beckman, A63882). We constructed a library based on the enriched cDNA fragments, which were attached to the C1 beads, using KAPA Hyper Prep Kits (KK8504). We used the NEB U-shaped adaptor for ligation. Libraries were sequenced to generate 150-bp paired-end reads on an Illumina NovaSeq 6,000 platform.

RNA-Seq raw reads with 10% low-quality bases, adapters and artificial sequences (including UP1, UP2, and polyA sequences) introduced during the experimental processes were trimmed by in-house scripts. Next, the trimmed clean reads were aligned to the GRCh38 reference using Tophat2 (v2.1.0) with the default settings (Trapnell et al., 2009). Cufflinks (v2.2.1) was further used to quantify the transcription levels of annotated genes (Trapnell et al., 2010).

### 2.3 Hi-C library preparation and data preprocessing

Hi-C libraries were constructed according to previous studies (Rao et al., 2014). Briefly, the samples were cross-linked with 1% formaldehyde for 10 min at room temperature and quenched with 0.125 M glycine for 5 min. The cross-linked cells were subsequently lysed. Endogenous nucleases were inactivated with 0.3% SDS, and chromatin DNA was digested with 100 U of MboI (NEB), marked with biotin-14-dCTP (Invitrogen) and then ligated with 50 U of T4 DNA ligase (NEB). After reversing the cross-links, the ligated DNA was extracted with a QIAamp DNA Mini Kit (Qiagen) according to the manufacturer’s instructions. The purified DNA was sheared to 300- to 500-bp fragments and further blunt-end repaired, A-tailed and adaptor-added, followed by purification through biotin-streptavidin–mediated pull-down and PCR amplification. The resulting Hi-C library was quantified and sequenced on a BGI T7 platform with a 150 bp paired-end length.

We preprocessed the Hi-C sequence data using Juicer v1.22.01 (Durand et al., 2016b) with alignment to GRCh38 using BWA (v0.7.8) (Li and Durbin, 2010) to eliminate ambiguous chimeric and nonalienable read pairs, duplicates, and otherwise low-quality alignments. Invalid pairs, such as dangling-end, relegation and self-circle pairs, were typically discarded at this step. A contact matrix was constructed at the level of genomic bins in which a continuous linear genome was partitioned into a fixed size in intervals (i.e., 20 kb, denoted as 20 kb matrix resolution). A bin size greater than 80% (covered by 1,000 reads) was considered the optimal bin size. The resulting contact heatmap was normalized by iterative correction and eigenvector decomposition (ICE) and the Knight-Ruiz matrix-balancing approach (KR) (Imakaev et al., 2012; Knight and Ruiz, 2012) to remove intrinsic biases within the matrix, which was followed by quantile normalization using BNBC (version 1.0.0) to remove biases between samples with default parameters, as described in our previous study (Jin et al., 2023). The reproducibility between duplicates was calculated by a weighted sum of correlation coefficients using HiCRep (Zhu et al., 2017).

### 2.4 Genome-wide contact matrix construction

We divided the linear genome into a given matrix resolution (1 Mb bins and 500 kb) and counted the “observed” number of contacts between two loci (*i,j*) in all intrachromosomal contact matrices, defined as the number of base pairs between the centers of the two loci. The “expected” number of contacts between each pair of loci was estimated by multiplying the fraction of reads containing i with the fraction of reads containing j and by multiplying by the total number of reads. The correlation coefficient of contacts was computed by taking the observed contact number between locus i and locus j (M_ij_) and dividing it by this expected value for all interchromosomal loci excluding any intrachromosomal locus pairs. Pearson’s correlation matrix was computed from the observed/expected value for each intrachromosomal locus pair with every interchromosomal locus pair.

### 2.5 A/B compartment calling and chromosome status dynamics

Chromosome compartmentalization was determined by principal component analysis (PCA) of the normalized Hi-C contact matrix at 20 kb resolution (Lieberman-Aiden et al., 2009a). The positive and negative eigenvectors of the first component (PC1 value) typically represented the A (active) and B (inactive) compartments in the Hi-C data. The A-B index, which represents the comparative likelihood of a sequence interacting with A or B, was calculated by subtracting the A and B values (Rowley et al., 2017) from the total values. Centromeric regions were excluded in the A/B partitions because no chromatin interactions were identified by Hi-C in these regions. Sex chromosome sequences were also excluded from the PCA.

To identify switched A/B compartment regions of the genome involved in muscle development, we first identified regions with statistically significant variability in PC1 values using HOMER (Heinz et al., 2010). We then considered the regions showing changes in PCA values from positive to negative or *vice versa* between the two stages. This analysis enabled us to define the genome regions that change compartment status between two time points. We then conducted functional enrichment analysis of genes in these compartments.

### 2.6 Identification of topologically associated domains (TADs)

We used a normalized contact matrix (generated by the Knight-Ruiz algorithm and quantile method) at 20 kb resolution to call domains. The hidden Markov model (HMM) was used to calculate the directionality index (DI) and to detect regions with biased upstream and downstream chromatin interactions (Dixon et al., 2012). We compared the contact frequency between two loci separated by TAD borders (inter-TADs) with a genome-wide average with an insulation score (IS) and distinguished the genome as topological domains, boundaries, and unorganized regions (Crane et al., 2015). The enrichment of protein-coding genes (PCGs) and housekeeping genes around TAD boundaries (±500 kb) was characterized. To identify stage-specific TAD boundaries, we merged the centre positions of the boundaries in two samples and calculated the Spearman correlation coefficients of directionality between the two stages. To compare the variation in the interaction intensity between relatively stage-invariant TADs, the contact frequency between two loci within the same TAD (intra-TAD) in consensus TADs (Malik and Patro, 2019) was characterized by the domain score (D-score) (Krijger et al., 2016) and calculated by dividing all intra-TADs within the same TAD by the sum of intra- and inter-TADs on certain chromosomes. TADs were defined as having more than 70% of the TAD bins belonging to the A compartment. Differentially topologically associated domains (TADs) were identified by the TADCompare package in R software (Cresswell and Dozmorov, 2020). TADCompare detected TAD boundaries by selecting regions with TAD boundary scores above a certain threshold (1.5 by default).

### 2.7 Identification of putative promoter-enhancer interactions (PEI), loops and estimation of mediated gene expression regulation

Hi-C interaction frequencies were used to construct a normalized contact matrix (using the knight-Ruiz algorithm and quantile method) using Juicer v1.22.01 (Durand et al., 2016b) and to define promoter-enhancer interactions (PEIs) using PSYCHIC v1.0 (Rosa-Garrido et al., 2017) at 20 kb resolution. Based on the normalized contact matrix at 10 kb resolution, we used the methods for “Loop identification” as implemented in Juicer to identify the significant loop interactions (Durand et al., 2016b). Specifically, the normalized contact matrix was split into a smaller matrix that consisted of 20 Mb × 20 Mb bins and 10 Mb steps of overlapping length. The putative enhancer regions were bins that significantly interacted with the promoter region that ranged from 2000 bp upstream to 500 bp downstream of the TSS. High-confidence interaction pairs (FDR values <0.01 and interaction distances ≥15 kb) were defined as putative PEIs. Juicebox (Durand et al., 2016a) and HiGlass (Kerpedjiev et al., 2018) were used for visualization.

### 2.8 Real time PCR analysis

Real-time quantitative PCR (qPCR) was conducted using a 15 μL reaction mixture that included 1 μL of synthesized cDNA, 0.6 μL each of reverse and forward primers at a concentration of 5 μM for the respective genes, 5.3 μL of double-distilled H2O (ddH2O), and 7.5 μL of SYBR®Prime Ex Taq™ II reagent (manufactured by Takara, Dalian, China). The qPCR reactions were run in triplicate in individual wells with the following thermal profile: an initial denaturation step at 95 °C for 10 min, followed by 40 cycles consisting of 10 s at 95°C, 30 s at 56°C, and 45 s at 72°C, concluding with a final extension at 72°C for 10 min. Negative control reactions were set up without any template DNA. Primers were listed in Table 1. Standard curves were established using pooled cDNA samples to evaluate the efficiency of amplification, and the specificity of the PCR products was validated through melting curve analysis. The qPCR data were processed using the 2^−ΔΔCT^ method (Livak and Schmittgen, 2001).

**TABLE 1:** Real time PCR primer information.

Gene	Primer sequence (5′-3′)	Product length (bp)	Tm (°C)
*TGM2*	F:AGCACAGGAGACCAAGAGAC	156	60
	R:CTCTCTAAGACCAGCTCCTCG		60
*GAS2L1*	F:CTGCTCCTCCACTGCTCATC	169	60
	R:CCCCTCCTTTGTGCTTCGTA		60
*PUS7L*	F:TCCACTGAACCGAGGCACT	108	60
	R:AGTGCCATGAAATCCAACGTGA		60
*GAPDH*	F: TCG​GAG​TCA​ACG​GAT​TTG​GT	181	60
	R: TTC​CCG​TTC​TCA​GCC​TTG​AC		60

## 3 Results and discussion

### 3.1 Initial characteristics of chromosomal conformation in the GV and MII follicle granulosa cell genomes

**FIGURE 1:**
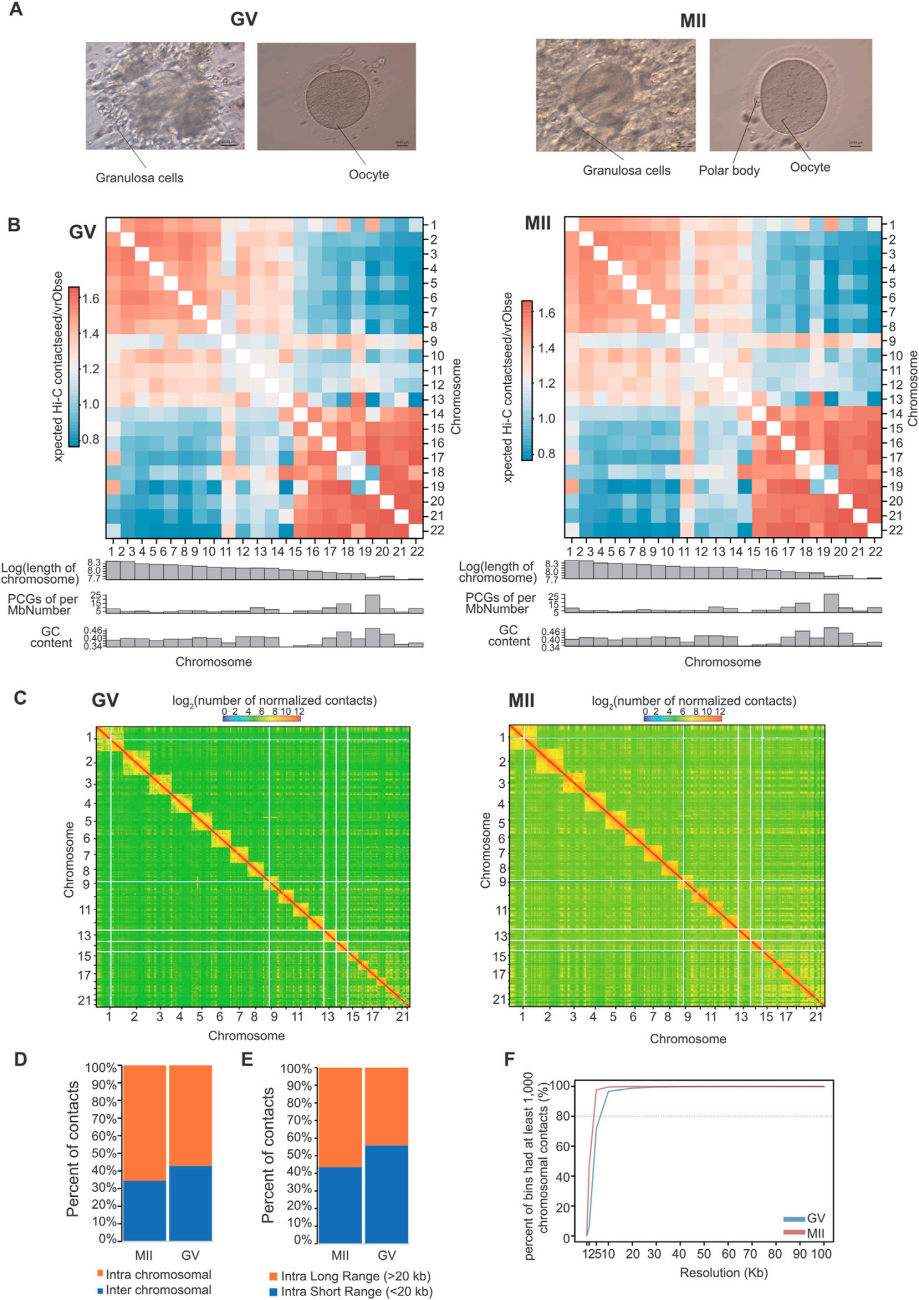
Overview of 3D organization in human GV and MII follicle granulosa cells. **(A)** Granulosa cells were sampled from GV and MII follicles. **(B)** Observed/expected contact matrices between chromosome pairs of GV (left panel) and MII (right panel) follicle granulosa cells. The length, gene density, and GC content of each chromosome are also shown. **(C)** Genome-wide contact maps indicated by normalized observed contact numbers of GV (left panel) and MII (right panel) follicle granulosa cells. **(D)** Percentage of *cis* (intra) and *trans* (inter) interactions among the Hi-C contacts. **(E)** Percentage of long contacts (>20 kb) and short contacts (<20 kb) of the cis interactions. **(F)** Resolution assessment of the *cis* contact matrix. The cumulative percentage of bins with 1,000 or more contacts is shown, along with the resolution bin size (1, 2, 5, 10, 20, 30, 40, 50, 60, 70, 80, 90 and 100 kb).

The maturation of oocytes has four important stages, namely, the germinal vesicle (GV) stage, germinal vesicle breakdown (GVBD) stage, metaphase I (MI) stage, and metaphase II (MII) stage (Lonergan and Fair, 2016; Strączyńska et al., 2022). To determine the dynamic changes in granulosa cell 3D chromatin organization mediated by gene expression during oocyte maturation, we performed Hi-C assays on granulosa cells from two of the stages, the GV and MII stages. A total of 450.93 Gb of data were generated (Table 2). These two stages represent well-characterized morphological and gene expression transitions that correspond to immature and mature oocyte formation, the critical period of oocyte maturation (Figure 1A).

**TABLE 2:** Data summary.

Sample	Read pair number	Base count (Gb)	Q20	Q30 (%)
GV	496,583,640	148.98	98.42%	93.67%
MII-1	97,952,892	149.39	97.21%	91.67%
MII-2	508,543,925	152.56	97.22%	91.83%

We then constructed genome-wide chromosomal contact maps by dividing the genome into 500 kb regions, revealing that human chromosomes have a similar likelihood of mutually contacting each other during development. Similar to other 3D genome studies (Li et al., 2022b; Zhang et al., 2022), the same type of chromosomes tended to be self-associated, small gene-rich chromosomes preferentially contacted each other more frequently, and more intensive interactions were observed within the same type of chromosomes than between types of chromosomes (Figures 1B, C). Widespread genomic contacts are generally associated with chromosomal ends, revealing the existence of chromosome territories (Gupta et al., 2015; Li et al., 2022b).

As a result, a total of 823.21 million significantly aligned contacts were generated by three Hi-C libraries. Of these contacts, ∼60.26% (496.09 million) were intrachromosomal contacts (Figure 1D) that were dominated by ∼49.03% long contacts greater than 20 kb (Figure 1E). Normalized intrachromosomal contact maps at 10 kb resolution (∼96.51% and 99.50% of bins had 1,000 or more reads for the GV and MII stages, respectively) were generated (Figure 1F).

### 3.2 Changes in the compartmentalization and local accessibility of GV and MII follicle granulosa cells

**FIGURE 2:**
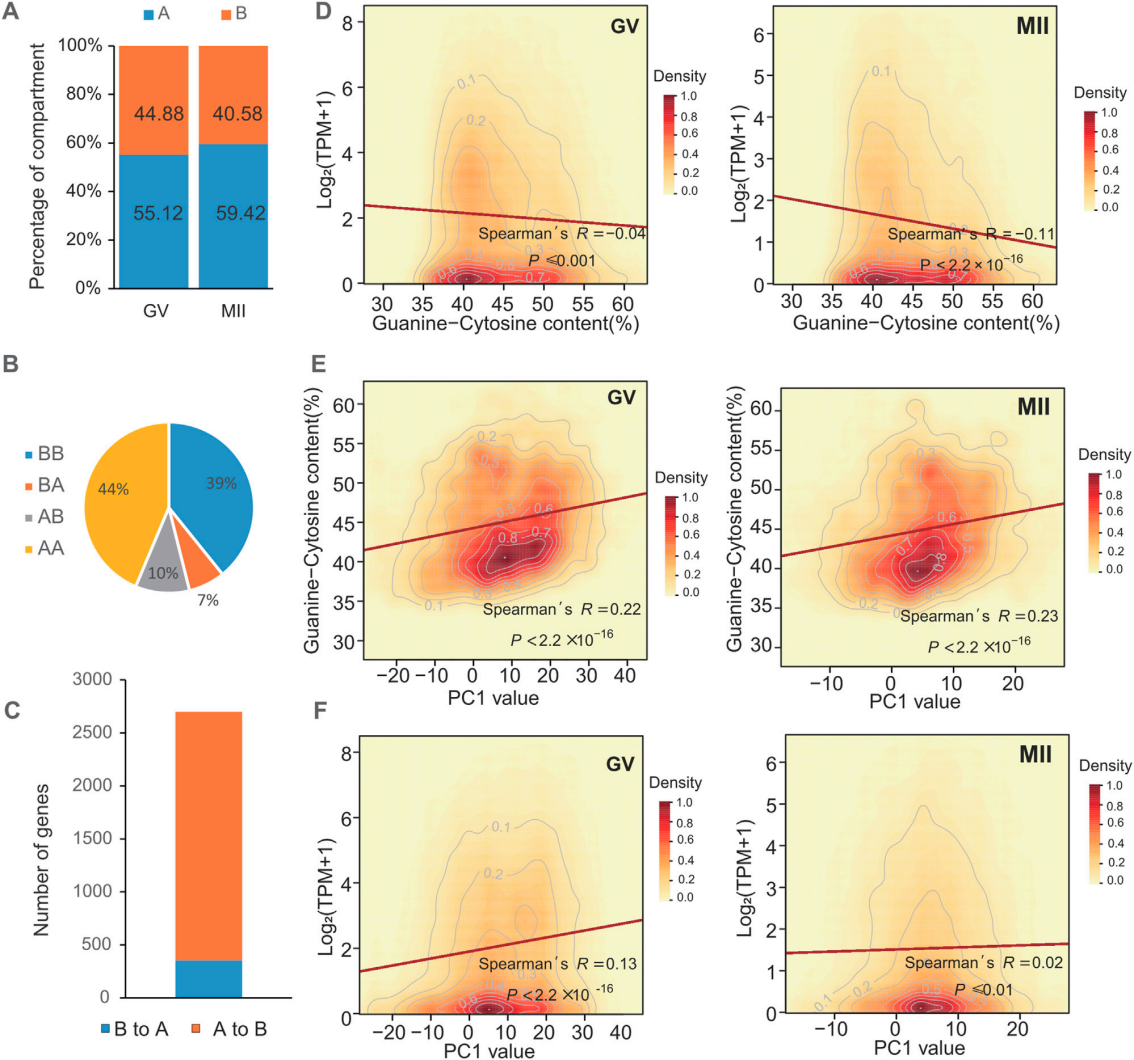
Compartmentalization dynamics in GV and MII follicle granulosa cells. **(A)** A/B compartment percentages of each stage. **(B)** A/B compartment percentages of stable and transitioned cells. **(C)** The number of genes in these transition regions. **(D)** The correlation between the expression levels of genes and GC content. **(E)** GC contents of A/B compartments. **(F)** Expression levels of genes located in A/B compartments.

At the compartment level, Pearson’s correlation between two intrachromosomal contact maps confirmed the findings of previous studies (Li et al., 2022b; Li et al., 2024). Each chromosome was divided into two types of regions (Figure 2A). One type is referred to as compartment A (which constitutes approximately 55.12% and 59.42% of the human GV and MII granulosa cell genomes, respectively). Among the compartment change regions, 75% were simultaneous stable genomic regions in the two periods, which were categorized as having stable (AA and BB) chromosome status (Figure 2B). From the GV stage to the MII stage, 17% of the human genome regions transitioned (Figure 2B), 10% from the A to B regions (2,346 embedded genes) and 7% from the B to A regions (353 embedded genes) (Figure 2C). For the whole genome, the expression levels of genes were negatively correlated with their GC content (Figure 2D). These compartment A regions were substantially enriched for the presence of a high GC content (Figure 2E) and for the highly expressed genes (Figure 2F). Displaying the opposite properties is compartment B, which is less accessible and constitutes 44.88% and 40.58% of the genomes. These known features provide an opportunity to explore the dynamic changes in compartmentalization patterns during oocyte maturation.

**FIGURE 3:**
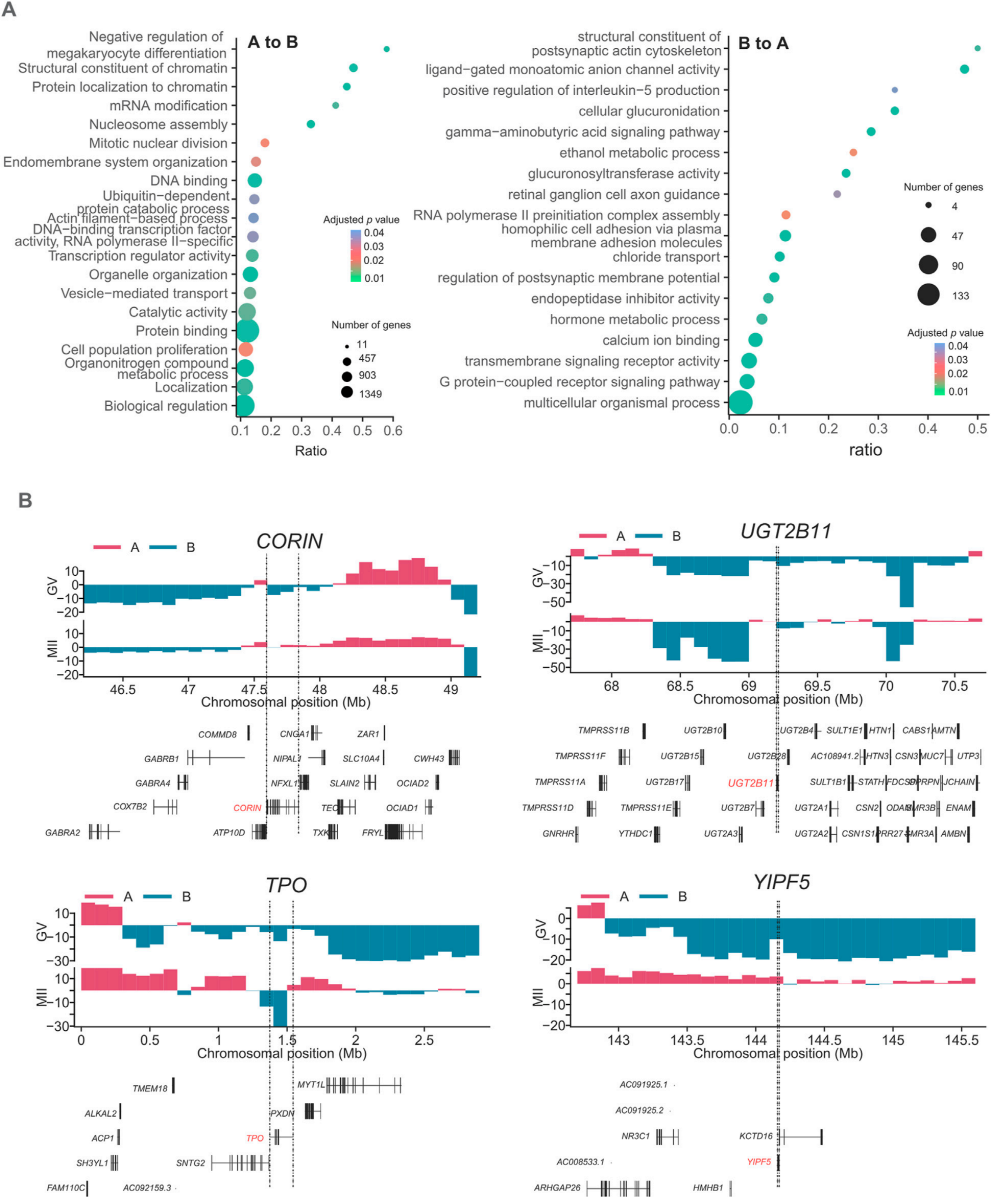
Features of compartments A/B in GV and MII follicle granulosa cells. **(A)** Gene Ontology (GO) and KEGG analyses of genes located in the A to B (left panel) and B to A (right panel) transition regions, respectively. **(B)** Four representative functional genes, namely, the *CORIN*, *UGT2B11*, *TPO*, and *YIPF5* genes, undergo compartment switching during skeletal muscle development. The dashed line boxes indicate the chromosomal locations of the genes.

Genes in regions A to B were particularly enriched in “vesicle-mediated transport”, “endomembrane system organization”, “cell population proliferation” and “mitotic nuclear division”. Moreover, genes in the B to A activated regions were enriched in “Regulation of postsynaptic membrane potential”, “Hormone metabolic process”, “ethanol metabolic process”, and “RNA polymerase II preinitiation complex assembly” (Figure 3A), reflecting functional gene activation from the GV stage to the MII stage. The *AKR1C3*, *TPO*, *SRD5A2*, *CORIN*, *UGT2B17*, *UGT2B7*, *UGT2B11*, *UGT2B28*, *UGT2B4*, *SULT1B1*, *SULT1E1*, *ADH4*, *ADH6*, *ADH1A*, *ENPEP*, and *YIPF5* genes are involved in “Hormone metabolic process”. These results indicate that during the development of oocytes from the GV to MII stages, hormone metabolic process-related pathways are enhanced. Corin is a transmembrane protease that processes natriuretic peptides (Yan et al., 2000). Atrial natriuretic peptide (ANP) is a hypotensive hormone converted from pro-ANP by corin and is involved in blood pressure homeostasis (Binder et al., 2023). Here, we found that the activation of *CORIN* is also related to oocyte maturation. *UGT2B11* is a member of the UGT2B subfamily, which consists of 11 members. The encoded UGT2B proteins are steroid-metabolizing enzymes with distinct but overlapping substrate specificities (Turgeon et al., 2001). Thyroid peroxidase (TPO) is essential for the synthesis of thyroid hormones (Sobitan et al., 2024). YIPF5 resides in the Golgi apparatus and is thought to play a critical role in vesicular trafficking (Pollin and Taylor, 2020). These genes all showed changes between compartment B and compartment A (Figure 3B). The gene expression of these four genes showed that only *CORIN* showed an increased expression (Supplementary Figure S1A).

### 3.3 TAD alterations are tightly coupled with transcriptional changes

**FIGURE 4:**
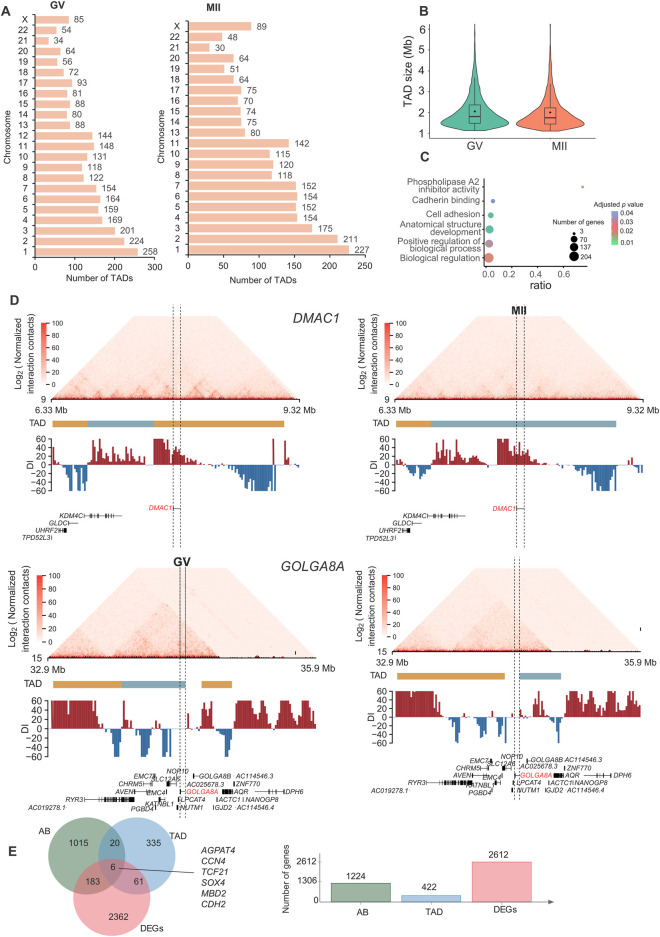
Characteristics and function of TADs in granulosa cells. **(A)** Number of TADs in each chromosome. **(B)** TAD size distribution in the GV and MII stages of granulosa cells. **(C)** Gene Ontology (GO) and KEGG analyses of genes in the differentiated TADs. **(D)** *DMAC1* (upper panel) and *GOLGA8A* (lower panel) in the relative TADs are shown. Top: Hi-C contact heatmaps of the genomic region around *DMAC1* and *GOLGA8A*. Bottom: Genome browser tracks of TAD locations and DI index signals. **(E)** Venn diagram showing genes with dynamic AB, TAD and expression changes.

A TAD is a distinct 3D chromatin domain with functional relevance to transcriptional regulation. Genomic loci within TADs encounter each other more frequently than do loci outside TADs (Dixon et al., 2012). TADs are conserved chromatin structures, which is consistent with previous reports (Yuan et al., 2021; Chen et al., 2022). We used the chromosome-wide insulation score (IS) and the directional index (DI) to identify TAD boundaries. We detected 2,787 and 2,440 TADs in GV and MII follicle granulosa cells (Supplementary Table S2), respectively (Figure 4A), with average lengths of 1.00 Mb and 1.05 Mb for the GV and MII stages, respectively (Figure 4B). We used Jaccard index to compare TADs between two stages, the jaccard index was 0.872.

Among the 3,642 TADs identified in the two stages, 430 were differentially TADs. Overall, most TADs identified in granulosa cells were stable during oocyte maturation. The genes in these differentiated TADs were mainly involved in “phospholipase A2 inhibitor activity”, “cadherin binding”, “anatomical structure development”, “cell adhesion”, “biological regulation”, and “positive regulation of biological process” terms (Figure 4C). The top differentially expressed genes in the TADs were *FAM53A*, *BCHE*, *GOLGA8A*, *CDH6*, *DMAC1*, *MCTP1*, *CNTNAP4*, *CT45A9*, *DTX2*, and *SLC39A11* (Supplementary Figures S2, S3); among these genes, *DMAC1* and *GOLGA8A* exhibited changes in the TAD structure (Figure 4D). Distal membrane arm assembly component 1 (DMAC1) was identified as an N-myristoylated protein that specifically localizes to mitochondria and plays critical roles in the assembly of complex I of the mitochondrial respiratory chain (Harada et al., 2023). Previous findings similarly show that *GOLGA8* is highly localized to the trans-Golgi and readily detectable at the plasma membrane, and overexpression of *GOLGA8* increased the activity of both splice modulation and RNase H1-dependent antisense oligonucleotides (McMahon et al., 2023). We next checked the genes with both dynamic compartment, TAD and gene expression change. As a result, we found that 6 genes were accompanied by all three changes. These genes were *AGPAT4*, *CCN4*, *TCF21*, *SOX4*, *MBD2*, and *CDH2* (Figure 4E). A recent study found that TCF3 and TCF12 are essential regulators in the process of oogenesis. A deficiency in these factors can negatively affect the activation of critical oocyte genes and the overall folliculogenesis (Liu et al., 2024), *TCF21* is also a member of this transcription factor family. Its function in folliculogenesis needs further in depth study.

### 3.4 Global rewiring of PEIs underpins functional divergence in granulosa cells during oocyte maturation

The regulatory specificity of enhancers and their interaction with gene promoters is thought to be controlled by their sequence and the binding of transcription factors (Kragesteen et al., 2018). To investigate the regulatory potential of enhancers on oocyte maturation-related genes, we identified a set of putative promoter-enhancer interactions (PEIs) (101,717 in the GV stage and 72,086 in the MII stage) that were assigned to substantially expressed genes (16,324 in the GV stage and 14,940 in the MII stage) (Figure 4A) at 20 kb resolution (adjusted *P*-value <0.05) (Supplementary Table S3).

**FIGURE 5:**
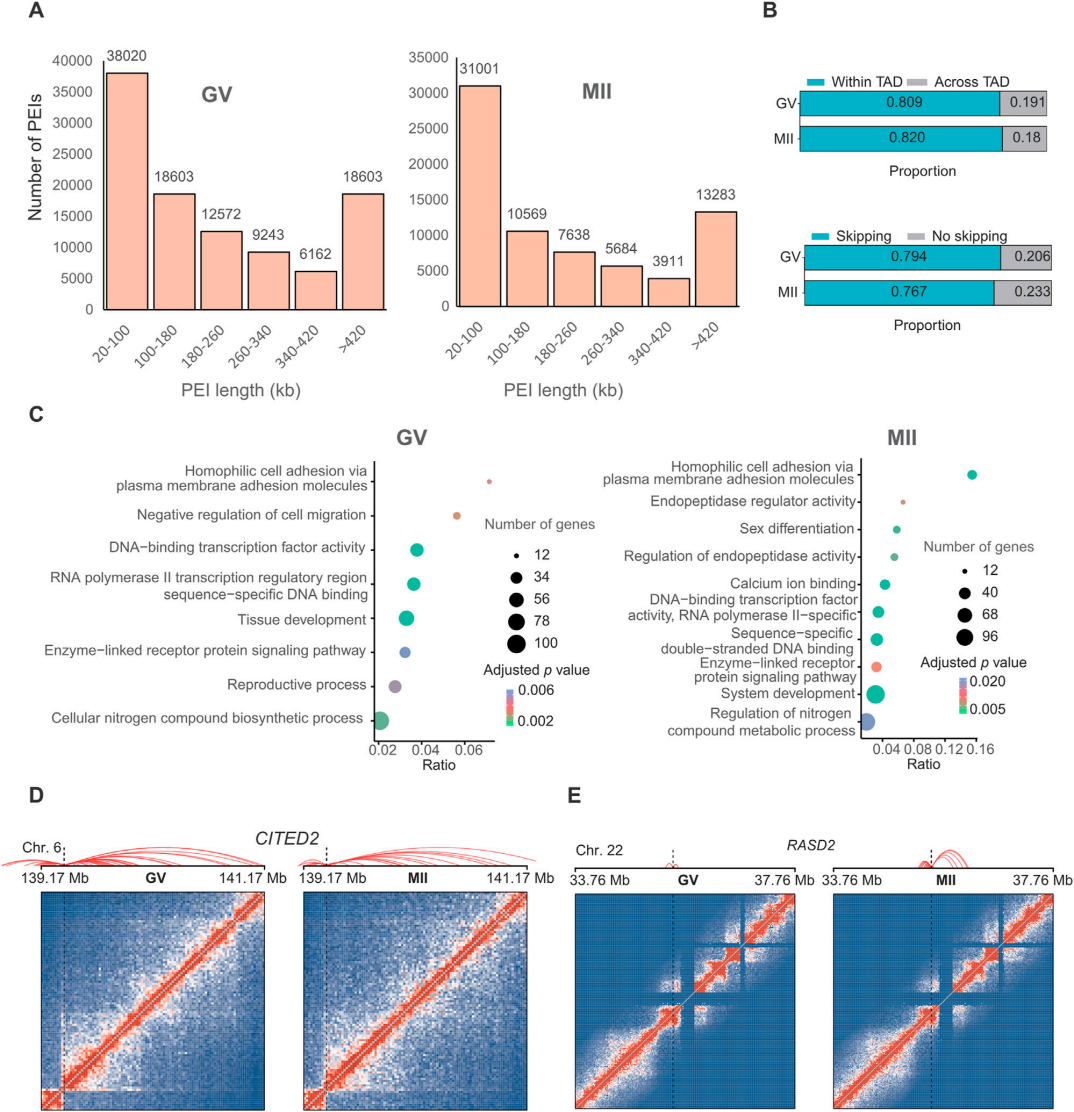
Promoter–enhancer interactions (PEIs) are rewired in granulosa cells during oocyte maturation. **(A)** Number of PEIs distributed in each stage. **(B)** Proportion of PEIs located within or across TADs (upper panel) and percentages of enhancers interacting with the nearest promoters (lower panel). **(C)** Gene Ontology (GO) and KEGG analyses of genes with the 300 genes with the greatest number of PEIs in the GV (left panel) and MII (right panel) stages. PEI rewiring of functional *CITED2* **(D)** and *RASD2* **(E)** in granulosa cells during oocyte maturation. Top: Schematic of PEIs and Hi-C contact heatmaps of the genomic region containing *CITED2* and *RASD2*. Middle: RNA-seq signals. Bottom: regional gene structures. The dashed line boxes indicate the chromosomal locations of the genes.

Approximately 36.84% of these contacts were short-range, i.e., within 100 kb (Figure 5A). We observed that 79.4% and 76.7% of putative enhancers skipped the neighboring promoter and interacted with distal target gene promoters in the GV and MII stages, respectively, and 80.9% and 82.0% of PEIs were constrained within TADs for the GV and MII stages, respectively (Figure 5B).

We further analyzed the genes with the greatest number of PEIs. In GV granulosa cells, we found that the 300 genes with the most PEIs were enriched in the “DNA-binding transcription factor activity”, “RNA polymerase II transcription regulatory region sequence-specific DNA binding”, “tissue development”, “cellular nitrogen compound biosynthetic process”, “negative regulation of cell migration”, “homophilic cell adhesion via plasma membrane adhesion molecules”, “reproductive process”, and “enzyme-linked receptor protein signalling pathway” terms (Figure 5C). In MII granulosa cells, we found that the 300 genes associated with the most PEIs were associated with the terms “calcium ion binding”, “sequence-specific double-stranded DNA binding”, “DNA-binding transcription factor activity, RNA polymerase II-specific”, “endopeptidase regulator activity”, “system development”, “homophilic cell adhesion via plasma membrane adhesion molecules”, “sex differentiation”, “regulation of endopeptidase activity”, “enzyme-linked receptor protein signalling pathway”, and “regulation of nitrogen compound metabolic process” (Figure 5C). Nine genes (*CITED2*, *OSR1*, *BCL2L11*, *HOXD13*, *TGFB2*, *ADAMTS1*, *NPPC*, *SFRP2*, and *FOXC1*) were involved in both the “reproductive process” of the GV stage and “sex differentiation” of the MII stage. These genes all had many PEIs in both stages. For example, *CITED2* had the greatest number of PEIs in the two stages (Figure 5D). A previous study revealed that the direct hsa-miR-548ba potential secondary target *BCL2L11*, hsa-miR-548ba, is involved in the regulation of follicle growth and activation via *BCL2L11* (Rooda et al., 2020). Transforming growth factor-β2 (*TGFB2*) is primarily expressed in oocytes, and its membrane receptors are located in cumulus cells; this gene is involved in expansion-related gene expression and consequent cumulus expansion (Hao et al., 2022). A Disintegrin and Metalloproteinase with Thrombospondin Motifs (ADAMTS), such as *ADAMTS1*, 4, 5 and 9, are enzymes that degrade proteoglycans in the extracellular matrix (ECM) of the follicles such that the oocytes can be released and regulate follicle development during folliculogenesis, favouring the action of essential growth factors, such as FGF-2, FGF-7 and GDF-9 (Hernández-Delgado et al., 2023).

We next analysed the top ten genes with the greatest decrease in the number of PEIs in the MII stage compared with those in the GV stage, which included *FAT3*, *PPP1R12C*, *DNAJC1*, *RPL28*, *TMEM238*, *PTPRH*, *COL12A1*, *IGFBPL1*, *ZNF581*, and *ZNF784*. The ten genes with the greatest increase in the number of PEIs in the MII stage included *VN1R2*, *ZNF677*, *ZNF813*, *DPRX*, *RASD2*, *CASP14*, *TC2N*, *HDGFL3*, *CCDC182*, and *KIF26B*. Most of these genes showed a higher expression in GV stage (Supplementary Figure S1B). Interestingly, *PARP12* forms granular aggregates near spindle poles during metaphase I (MI) and metaphase II (MII) (Cao et al., 2023). *PARP12* depletion results in abnormal spindle organization and chromosome misalignment in mouse oocytes (Cao et al., 2023). *RASD1* is a novel factor in the MI-MII oocyte transition and may be involved in regulating the progression of cytokinesis and spindle formation, controlling related signalling pathways during oocyte maturation (Lee et al., 2016). The functions of the *PPP1R12C* and *RASD2* genes need further in-depth study to explore their roles in oocyte maturation. *RASD2* had more PEIs in the MII stage (Figure 5E). KIF26B is a member of the kinesin family (KIF) that is composed of 2,108 amino acids, and recent studies have suggested that *KIF26B* plays an important role in the oncogenesis or progression of many human cancer types (Wang et al., 2021). Here, we found that *KIF26B* also plays an important role in oocyte maturation. We also detected the loops in these two stages, as a result, only 493 and 23 loops were identified in GV and MII stages, respectively. This limited number of loops identification might be caused by only ∼50 and ∼100 folds coverage of genome for each stage were sequenced, and loop identification need at least 5 kb resolution for accurate identification. Thus, we did not further analyze their difference. To verify the consistency of gene expression, we randomly selected three genes for qPCR analysis, the results suggest that the expression between RNA-seq (Supplementary Table S4) and qPCR are similar (Supplementary Figure S1C).

Understanding 3D genome organization in granulosa cells is crucial for deciphering the regulatory networks that govern oocyte development. The insights gained from our study may have implications for reproductive health, as aberrations in 3D genome organization could contribute to infertility or other reproductive disorders. Furthermore, this knowledge could inform the development of novel strategies for assisted reproductive technologies, thus enhancing their success rates. By applying these techniques to granulosa cells from GV and MII follicles, researchers can further elucidate the spatial organization of the genome of granulosa cells at different stages of follicular development.

## 4 Conclusion

In conclusion, the investigation of 3D chromatin organization in granulosa cells from GV and MII follicles has elucidated dynamic changes that occur during oocyte maturation. Our study revealed distinct compartmentalization patterns, highlighting the prevalence of stable genomic regions and transitions from one compartment to another. Notably, functional gene activation and hormonal metabolic processes were found to be enriched during the transition from the GV to MII stage, indicating significant functional divergence during oocyte maturation. Additionally, we identified a subset of genes with altered promoter-enhancer interactions (PEIs), indicating a regulatory shift in gene expression related to crucial reproductive processes. These findings help characterize the intricate regulatory networks governing oocyte development, potentially offering valuable insights for reproductive health and the development of innovative strategies for assisted reproductive technologies. By examining the spatial genome organization of granulosa cells at different follicular development stages, this research may lead to a deeper understanding of the molecular mechanisms underpinning oocyte maturation and their implications for fertility and reproductive disorders.

## Data Availability

The datasets presented in this study can be found in online repositories. The names of the repository/repositories and accession number(s) can be found below: https://www.ncbi.nlm.nih.gov/, PRJNA1116765.

## Supplementary material

SUPPLEMENTARY FIGURE S1 The mRNA abundance. **(A)** The mRNA abundance for RNA-seq (left panel) and qPCR (right panel) results in different stages. **(B)** The mRNA abundance for genes with AB dynamic change in Figure 3B. **(C)** The mRNA abundance for genes with TAD changes in Figure 4D; Supplementary Figures S1, S2.

SUPPLEMENTARY FIGURE S2
*FAM53A*, *MCTP1*, *DTX2*, and *SLC39A11* in the relative TADs are shown. Top: Hi-C contact heatmaps of the genomic region around each gene. Bottom: Genome browser tracks of TAD locations and DI index signals.

SUPPLEMENTARY FIGURE S3
*BCHE*, *CDH6*, *CNTNAP4* and *CT45A9* in the relative TADs are shown. Top: Hi-C contact heatmaps of the genomic region around each gene. Bottom: Genome browser tracks of TAD locations and DI index signals.
