# Supplementary figures and images for: Comparative study of the three-dimensional genomes of granulosa cells in germinal vesicle and metaphase II follicles
Source: Front Genet. 2024 Nov 20;15:1480153. doi: 10.3389/fgene.2024.1480153 (PMC11615058; doi:10.3389/fgene.2024.1480153)

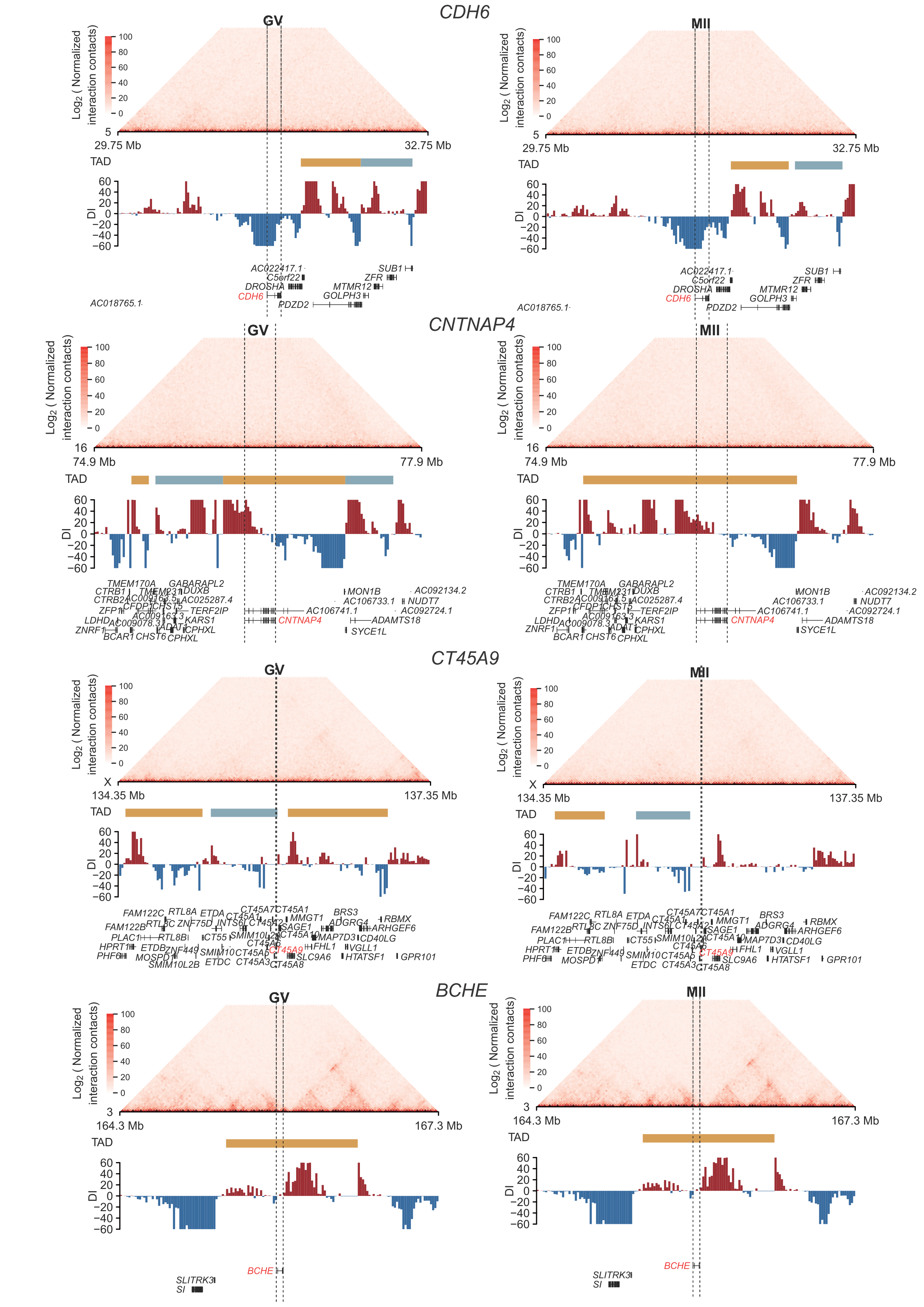

Supplement: Supplementary file 1 [file Image3.TIF]

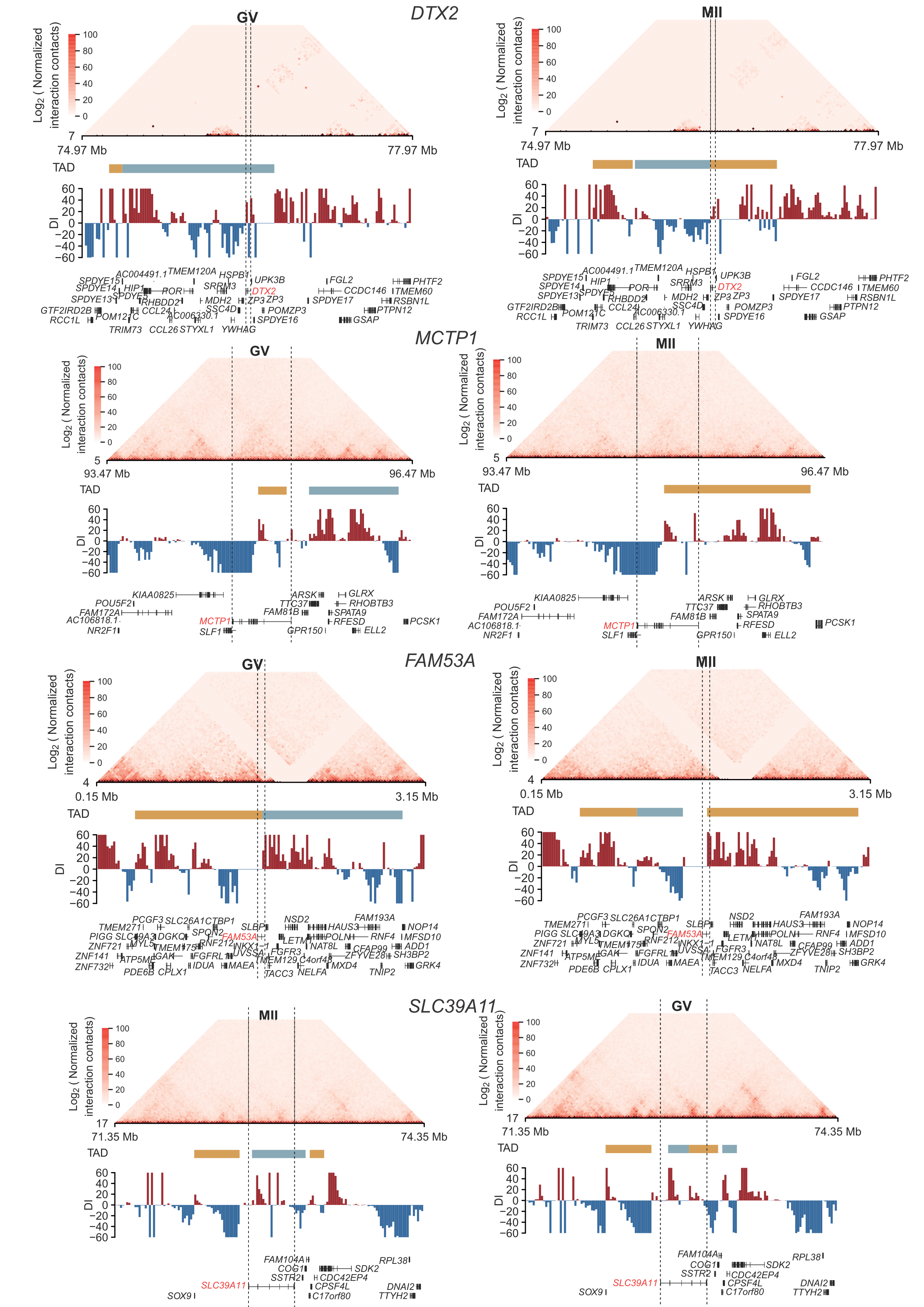

Supplement: Supplementary file 2 [file Image2.TIF]

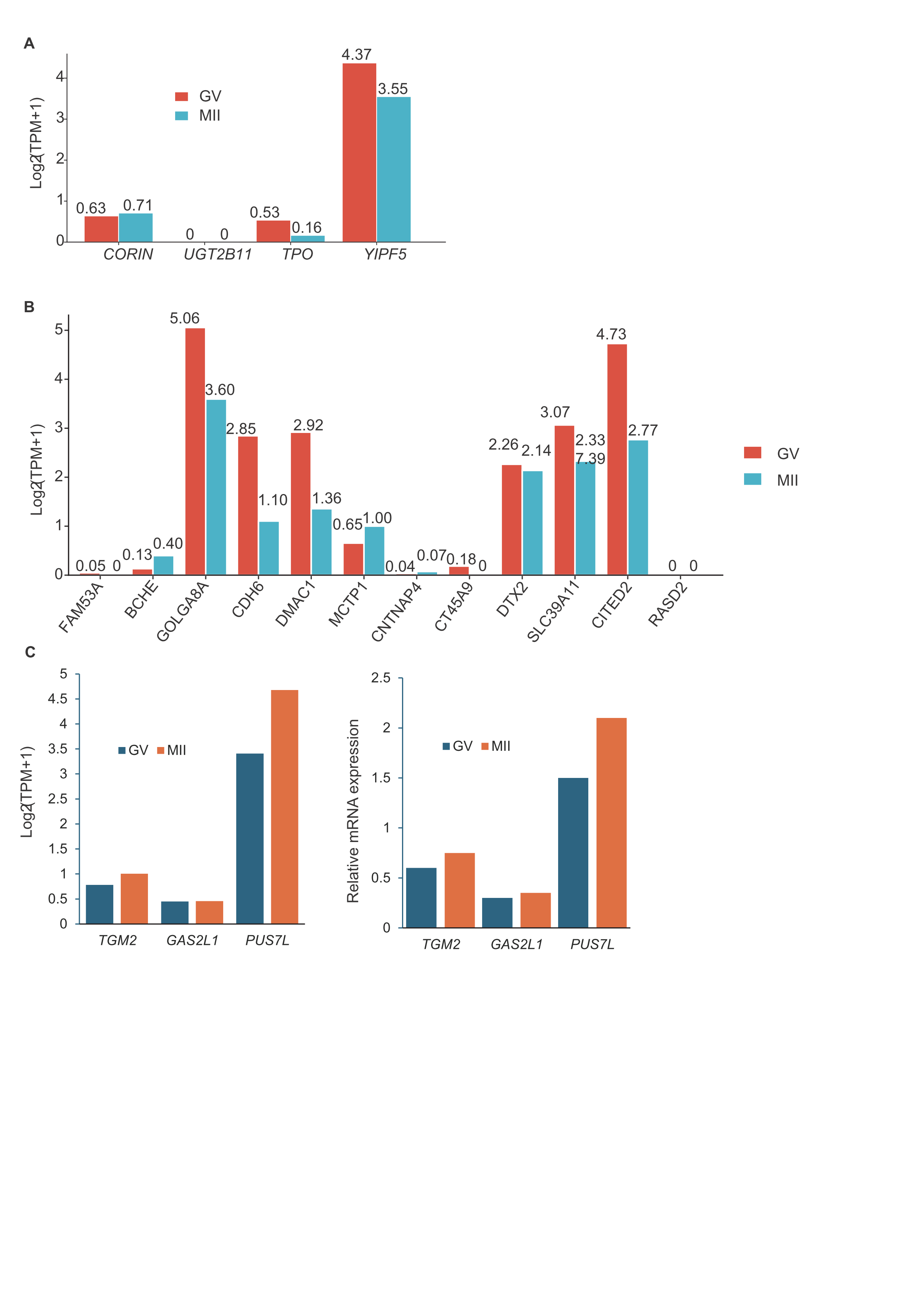

Supplement: Supplementary file 3 [file Image1.TIF]
